# Supplementary material for: Coping strategies and changes in type D personality were associated with depressive tendency at 9 months after percutaneous coronary intervention
Source: PLoS One. 2025 Jan 13;20(1):e0316639. doi: 10.1371/journal.pone.0316639 (PMC11729923; doi:10.1371/journal.pone.0316639)
Supplement: Attachment — Submitted filename: Response to Reviewers.docx [file pone.0316639.s005.docx]

**Response to Reviewers**

Thank you for reviewing our manuscript. We have revised the manuscript in accordance with the feedback provided.

**Response to Reviewer 1's review comments**

Thank you very much for your valuable comments. We have made the corrections as suggested and requested the translation company Editage to proofread the English. Please find our responses to your comments and the revised text below.

**Abstract**

*#1. Please define the origin of Type D personality shortly*

Response: Thank you for your comment. We added the following sentence to the top of the abstract.

(Line 28) Type D personality, characterized by negative affectivity and social inhibition, has been associated with both the psychophysiology of coronary artery disease (CAD) and depressive disorders. However, few reports have described the impact of coping strategies in these patients.

*#2. Line 39: tendency was observed in patients*

Response: We fixed this.

*#3. Line 41, 43, 45: with the adoption of*

Response: We fixed this.

*#4. Line 45: Changes in type D personality*

Response: We fixed this.

*#5. A closing sentence after the results is missing (Conclusion).*

Response: We agree with you and have added the following sentence:

(Line 47) Personality-oriented treatment incorporating specific coping strategies may provide new strategies to prevent depression and improve care for patients with CAD.

**Introduction**

*#6. The definition of a type D personality is missing. This should be integrated.*

Response: Thank you for your important comment. The definition of a type D personality is revised as follows.

(Line 66) Type D (distress) personality, characterized by negative affectivity and social inhibition, has emerged as a predictor of CAD onset. Negative affectivity involves the suppression of emotional expression, while social inhibition involves suppressing emotional and behavioral expression (10). Furthermore, Type D personality has been identified as a factor associated with depression (11).

*#7. Line 53: Which strategies are appropriate? Add some examples.*

Response: Thank you for your question. We deleted the confusing phrase and added the importance of care and treatment for depressive patients with CAD by adding 2 references as follows.

(Line 56) Psychological care and treatment are necessary for patients with CAD and depression (6); however, only 4.1% of them receive antidepressant treatment (7).

Additional references:

6.	Thombs BD, de Jonge P, Coyne JC, Whooley MA, Frasure-Smith N, Mitchell AJ, et al. Depression screening and patient outcomes in cardiovascular care: A systematic review. JAMA. 2008;300: 2161-2171. doi: [10.1001/jama.2008.667](https://doi.org/10.1001/jama.2008.667).

7.	Peltzer S, Müller H, Köstler U, Schulz-Nieswandt F, Jessen F, Albus C. CoRe-Net study group. Detection and treatment of mental disorders in patients with coronary heart disease (MenDis-CHD): A cross-sectional study. PLOS ONE. 2020; 15;8: e0243800. *Line 56: aged 55 years and older*.

　Response: We fixed this.

*#9. Line 68: What is the meaning of "pathological condition"?*

　Response: Thank you for your question. The meaning was confusing, and the sentence was revised as follows.

(Line 71) The prevalence of Type D personality in patients with CAD has been reported as follows: 30% in those with angina pectoris, 28% in those with myocardial infarction, and 31% in those with ischemic heart failure (12).

*#10. Line 70: The percentages of healthy individuals who have type D personality are quite similar compared to the percentages of clinical samples which you have mentioned in the sentence before. This should be highlighted.*

Response: As you pointed out, similar percentages of type D personality were observed in healthy individuals and in CAD patients. In accordance with your suggestion, we highlighted this by adding the following sentence.

(Line 75) The similarity in Type D personality prevalence between patients with CAD and healthy individuals suggests that the prevalence of this personality trait may not be dependent on the presence of CAD.

*#11. Line 74: Why 55.9 % and not 56 % (summing up to 100 %)?*

Response: The results were presented with the first decimal place. We reconfirmed the calculation, and the result was correct. As the sentence was confusing, we simplified the text as follows:

(Line 80) A particularly high rate (55.9%) of type D personality was found among those with depressive tendencies.

*#12. Line 72: Are the assessment tools for depressive tendencies the same or different? Name the questionnaires.*

Response: We used the same Self-rating Depression Scale (SDS) in this study and our previous studies (references 13 and 14). Therefore, we added the name of this scale on line 79.

*#13. Line 105: Which kind/version of STROBE checklist do use?*

Response: The STROBE checklist used is version 4 for cross-sectional studies (Line 113).

*#14. The sentences with findings of other studies regarding Odds Ratio should be written less technically and more vivid.*

*The paragraphs in the introduction part could be linked more smoothly.*

Response: Thank you for your valuable comments. We deleted odds ratios in lines 61, 62, 94, 95, 313, 351 and 352.

In addition, we simplified the sentences that contained odds ratios as follows:

　(Line 69) Furthermore, type D personality has been identified as a factor associated with depression .

(Line 84) Among patients with depressive tendencies, 59.2% had a type D personality, indicating a high association at one month after discharge.

Some odds ratios were preserved in the text, which were considered important to compare with results of our previous studies.

**Methods - Measurements**

*#15. Reasons for the questionnaires used should be briefly stated.*

Response: We have provided the reasons for the use of questionnaires in this study.

(Line 154) This scale, which has been used to evaluate depressive tendencies in clinical studies of

cardiovascular disease (31,32)...

(Line 163) This scale evaluates three categories of coping strategies: the problem-focused/emotion-focused axis, approach-avoidance axis, and cognitive-behavioral axis.

Additional references:

31.	Kala P, Hudakova N, Jurajda M, Kasparek T, Ustohal L, Parenica J, et al. Depression and anxiety after acute myocardial infarction treated by primary PCI. PLOS ONE. 2016;11: e0152367. doi: [10.1371/journal.pone.0152367](https://doi.org/10.1371/journal.pone.0152367).

32.	Wang L, Mei F, Min M, He X, Luo L, Ma Y. Adoption of the cardiopulmonary exercise test in the exercise ability and cardiopulmonary function rehabilitation of coronary artery disease (CAD) patients. BMC Cardiovasc Disord. 2024; 21;16: 313. doi: [10.1186/s12872-024-03958-0](https://doi.org/10.1186/s12872-024-03958-0)*.*

*#16. Example items could be integrated for a better understanding.*

*The coping strategies could be executed more.*

Response: Thank you for your advice. We realize that our explanation about evaluation of Tri-axial Coping Scale was insufficient. We have replied to your comment #18 by adding an explanation about coping strategies with an additional reference, so please see our response to #18.

*#17. Figure 1: "Patients with missing values" are excluded. Why do not use imputation as a statistical method?*

Response: The 9 months follow-up questionnaires were collected by mail, which led to random missing values. The exclusion of these missing responses had minimal impact on the analysis. Also, the sample size for the logistic regression analysis remained adequate after excluding incomplete responses. Therefore, we considered the removal of missing values acceptable for this analysis.

*#18. Line 150 Coping strategies: Is it a self-administered questionnaire? What does tri-axial mean?*

Response: Yes, this is a self-administered questionnaire. The term ‘Tri-axial’ refers to a classification system of coping strategies along three dimensions: the "problem-focused/emotion-focused axis," "approach-avoidance axis," and "cognitive-behavioral axis." Since the previous explanation was insufficient, the text has been revised to clarify this.

(Line 162) Coping strategies were assessed using the Tri-axial Coping Scale (TAC-24), a self-administered questionnaire (35). This scale evaluates three categories of coping strategies: the problem-focused/emotion-focused axis, approach-avoidance axis, and cognitive-behavioral axis (36).

Additional reference:

36.	Tatsuno J, Yamase H, Yamase Y. Grief reaction model of families who experienced acute bereavement in Japan. Nurs Health Sci. 2012;14: 257-264. doi: 10.1111/j.1442-2018.2012.00688.x.

*#19. Line 177: You use several statistical methods but the sample size is quite low. Therefore, some calculations could be only exploratory. Please, mention the problem of multiple testing.*

Response: As the reviewer pointed out, a major limitation of this study is the small sample size. The manuscript has been revised as follows.

(Line196) The study included 18 patients with improved depressive tendencies at 9 months and 29 with continued depressive tendencies. Due to the small sample size, logistic regression analyses were exploratory.

(Line 183) For comparisons involving three or more variables, Bonferroni correction was performed applied to account for multiple comparisons.

**Results**

*#20. Line 189: Are there any sociodemographic or other differences between respondents with complete values and with missing values?*

Response: When we compared the baseline characteristics between 84 patients with complete values and 25 patients with missing values, no variable showed significant differences. We have included a table to show this.

|  | Missing values  (n=25) | Complete values (n=84) | p |
| --- | --- | --- | --- |
| Baseline characteristics |  |  |  |
| Age | 64.0 [53.5–72.0] | 66.0 [58.0–72.8] | 0.571 |
| Male / female | 21 (84.0)/14(16.0) | 75 (89.3)/ 9 (10.7) | 0.474 |
| Living with family / alone | 20 (80.0)/5 (20.0) | 77 (91.7)/7 (8.3) | 0.102 |
| Employed / unemployed | 12 (48.0)/13 (52.0) | 52 (61.8)/32 (38.1) | 0.215 |
| Type D personality | 13 (52.0) | 34 (40.5) | 0.307 |
| Acute myocardial infarction | 12 (48.0) | 29 (34.5) | 0.222 |
| Hypertension | 15 (60.0) | 52 (61.9) | 0.864 |
| Dyslipidaemia | 18 (72.0) | 63 (75.0) | 0.763 |
| Diabetes mellitus | 11 (44.0) | 27 (32.1) | 0.275 |
| Body mass index (kg/m^2^) | 23.5 [21.5–27.5] | 24.3 [21.7–27.1] | 0.692 |
| Depressive tendency | 19 (72.5) | 47 (56.0) | 0.072 |
| Subscales of the tri–axial coping scale |  |  |  |
| Catharsis | 8.0 [6.0–11.0] | 8.0 [6.0–11.0] | 0.980 |
| Abandonment or resignation | 7.0 [5.5–8.0] | 7.0 [6.0–9.0] | 0.959 |
| Information gathering | 8.0 [7.0–12.0] | 9.0 [7.0–11.0] | 0.523 |
| Distraction | 8.0 [6.0–11.0] | 8.5 [7.0–10.0] | 0.279 |
| Evasive thinking | 9.0 [7.0–10.5] | 9.0 [7.0–10.0] | 0.120 |
| Positive interpretation | 11.0 [10.0–13.0] | 10.0 [8.5–12.0] | 0.185 |
| Planning | 10.0 [8.0–12.0] | 10.0 [9.0–13.8] | 0.640 |
| Responsibility shifting | 4.0 [3.0–6.0] | 4.0 [3.0–6.0] | 0.709 |

Baseline characteristics were compared by the Mann-Whitney U test for continuous variables and the χ^2^ test for nominal variables.

*#21. Table 1: Have you informations about the status as past smoker?*

Response: No, we did not investigate past smoking. We agree with the reviewer's concern, because there have been inconsistent results of association between smoking and depression (Fluharty M, Taylor AE, Grabski M, Munafò MR. The association of cigarette smoking with depression and anxiety: a systematic review. Nicotine Tob Res. 2017;19: 3-13.). This is our interest to be investigated in the future.

*#22. Why do you take creatinine kinase? Dyslipidemia is quite general. Do you have further informations? If no, mention this a limitation of the study.*

Response: We investigated the peak levels of creatinine kinase and BNP, which represented the severity of myocardial infarction and heart failure, respectively. Since we did not have further information regarding dyslipidemia or diabetes, we have mentioned this as a limitation, following the reviewer's comment.

(Line 407) Fifth, there was no detailed data on dyslipidemia or diabetes.

*＃23. Line 199: "frequency of depressive tendency" Please, clarify in this case and other cases the measurement point (baseline, 9 months later or both?).*

Response: We have revised the sentence to clarify this.

(Line 217) At the 9-month evaluation, the prevalence of depressive tendency and type D personality was 47.6% and 40.5%, respectively.

*#24. Line 204: "abandonment or resignation" For the discussion section: What is coping and what is a symptom of depression? Discuss it.*

Response: We have added the explanation and discussion of depressive symptoms, coping, and "abandonment or resignation" to the following lines.

(Line 60) time-dependent depressive symptoms, such as depressed mood, insomnia, loss of appetite.

(Line 87) One effective intervention to address depressive tendencies is the implementation of coping strategies, which are cognitive and behavioral efforts to reduce stress (16). Research has shown that individuals who do not use effective coping strategies are more likely to experience depressive symptoms (16,18).

Additional reference:

18.	Tantrarungroj T, Ocharoen P, Sachdev V. Grief reaction, depression, anxiety, and coping of relatives after palliative patients' death in Thailand. PLOS ONE. 2022;17: e0276583. doi: [10.1371/journal.pone.0276583](https://doi.org/10.1371/journal.pone.0276583).

(Line 163) This scale evaluates three categories of coping strategies: the problem-focused/emotion-focused axis, approach-avoidance axis, and cognitive-behavioral axis.

(Line 326) Patients using the "abandonment or resignation" strategy may avoid confronting reality and passively escape from stress, leading to a higher likelihood of depressive tendencies (15).

*#25. Subheading – Patients: sometimes patients/participants -> considerate using one term*

*Sometimes it is about the depressive tendency of participants sometimes that participants are depressive. This is a difference. According to the introduction the study is about depressive tendency. The parts writing about depressive patients/participants should be adapted. This should also be taken into account in table 2: depressive - non-depressive. There is no correction for multiple testing (like Bonferroni correction).*

Response: These expressions have been unified to "patient" and "depressive tendency".

Response: In line 183-185, we mentioned that "For comparisons involving three or more variables, Bonferroni correction was applied to account for multiple comparisons."

*#26. Line 230: Where is the assessment (statistical data) of this missing correlation?*

Response: We have added the data to Line 240 "No association was observed between depressive tendencies at 9 months and baseline type D personality: OR 1.43 (95% CI, 0.60–3.43)."

**Discussion**

*#27. Line 281: "planning strategy" at baseline or 9 months later. A figure regarding the different questionnaires at different measurement points could be helpful.*

Response: We have added the measurement points with questionnaire items to Figure 1.

*#28. No enumeration of outcomes and no reporting of outcomes (naming of OR), these should be removed and the sections reworded.*

Response: We have removed the ORs from the discussion section in lines 61, 62, 70, 85, 95, 313, 351, and 352.

*#29. A major limitation is multiple testing. This should be mentioned in the discussion.*

Response: This is an important limitation of this study. In addition to the description in the limitation section, we have revised the manuscript by adding further explanations in the methods and the discussion sections as follows:

(Line 372) However, due to the small sample size, further research is needed to investigate the individual associations of coping strategy subscales with personality traits and depressive tendencies.

(Line 197) Due to the small sample size, logistic regression analyses were exploratory.

*#30. Personality traits are quite stable over the life time. Please, discuss why type D behavior patterns should change within several months (trait vs. state). Furthermore, it is necessary to discuss the limitations of the type D concept.*

Response: Currently, there is no research demonstrating a change in type D personality. The SPIRR-CAD study (comment #32, new reference 48), reported a reduction in type D personality scores through stepwise psychological intervention. However, our study does not clarify why type D personality changed. It is possible that patients with a stress-prone type D personality experienced reduced stress after discharge by altering their lifestyle, which might lower the effects of stress hormones, such as cortisol and norepinephrine. Nonetheless, clear evidence for this is lacking. We have addressed the reviewer's comment and revised the manuscript accordingly.

(Line 386) Currently, there is limited evidence regarding psychological intervention that can change type D personality (57). However, a recent study has demonstrated that stepwise psychotherapy interventions can reduce the scores of type D personality scale (48). Since mechanisms underlying changes in type D personality were beyond the scope of this study, future research is required to elucidate these mechanisms.

Additional reference:

57.	Kupper N, Denollet J. Type D personality as a risk factor in coronary heart disease: A review of current evidence. Curr Cardiol Rep. 2018;20: 104. doi: [10.1007/s11886-018-1048-x](https://doi.org/10.1007/s11886-018-1048-x).

*#31. Some CI (see table 5) are quite near by 1.0 (0,5-0,99) re abandonment or resignation.*

*Discuss this, please.*

Response: We have added the following explanation to the legend of Table 5.

(Line 294) If upper or lower ranges of the 95% CI were close to 1.0, the significance of OR and its association should be considered as minimal.

*#32. Line 320: There is the the so-called SPIRR-CAD study on depressed CAD patients with and without type D personality traits with psychodynamic parts of the intervention.*

Response: We have added the SPIRR-CAD study to the reference list and revised the manuscript as follows:

(Line 368) Interestingly, stepwise psychotherapy may benefit depressed patients with CAD with type D personality, while such benefits were not observed in patients with non-type D personality (53). Thus, personality-specific interventions should be considered for patients with CAD.

Additional reference:

53.	Herrmann-Lingen C, Beutel ME, Bosbach A, Deter HC, Fritzsche K, Hellmich M, et al. A stepwise psychotherapy intervention for reducing risk in coronary artery disease (SPIRR-CAD): Results of an observer-blinded, multicenter, randomized trial in depressed patients with coronary artery disease. Psychosom Med. 2016;78: 704-715. doi: 10.1097/PSY.0000000000000332.

*#33*. *Line 339: the present study*.

Response: We have corrected this.

*#34. Some practical conclusions could be elaborated more specific.*

Response: We have revised the last sentence of the conclusion accordingly. We refrained from making further inferences or speculations in the conclusion section to maintain clarity and accuracy.

(Line 416) Therefore, personality-oriented intervention and/or education incorporating specific coping strategies may be beneficial in preventing and improving depression, which, in turn, could positively impact the prognosis of patients with CAD.

*#35. Figures in poor quality, therefore unreadable. Figure description in different font than text.*

Response: Following your instruction, we upload figure files to the Preflight Analysis and Conversion Engine (PACE) digital diagnostic tool, and then download tiff files. We have also adjusted the font-type of the figure descriptions to comply with the reviewer's comment and the formatting guidelines.

*#36. Table 4: Why median and not mean?*

Response: The Shapiro–Wilk test indicated that none of the baseline characteristic variables were normally distributed. Since the median is not affected by distribution, we standardized the results using the median.

(Line 185) Continuous variables of baseline characteristics are described as medians [four-part range] because the Shapiro–Wilk test demonstrated that the variables were not normally distributed.

*#37. Table 5: Numbers stand for models, not for literature. Please, clarify it.*

Response: We have revised the legend of Table 5 as follows:

(Line 291) Odds ratios (OR) and 95% confidence intervals (CI) for improvement in depressive tendencies were calculated for each subscale of the tri-axial coping scale, adjusted for baseline age in model 1, baseline sex in model 2, and baseline Type D personality in model 3.

***Response to Reviewer 2's review comments***

**Introduction**

*#38. The introduction appears to be a little bit confused with an extensive list of data that leads the reader to get lost in the reading. Then, type D personality and depressive tendency which should be the central constructs of the introduction with coping strategies are explained in a superficial manner (type D personality is explained in less than one sentence in line 64) without making the reader understand precisely what is being talked about.*

*I suggest restructuring the introduction, starting with a clear and comprehensible explanation of type D personality, depressive tendency and coping strategies (even if only with a general explanation, and then focusing in more detail on the specific coping strategies discussed later on); then continuing with all the literature data concerning the association between these personality 'styles', cardiovascular risk and coping strategies, and then concluding with the aims of the paper.*

*This will allow a more linear reading of the work.*

Response: We have integrated your comments along with Reviewer 1's comments #6 and #24 and added explanations regarding type D personality and coping strategies.

(Line 66) Type D (distress) personality, characterized by negative affectivity and social inhibition, has emerged as a predictor of CAD onset. Negative affectivity involves the suppression of emotional expression, while social inhibition involves suppressing emotional and behavioral expression (10). Furthermore, Type D personality has been identified as a factor associated with depression (11).

(Line 87) One effective intervention to address depressive tendencies is the implementation of coping strategies, which are cognitive and behavioral efforts to reduce stressors [16]. Research has shown that individuals who do not use effective coping strategies are more likely to experience depressive symptoms (16,18).

Additional reference:

18.	Tantrarungroj T, Ocharoen P, Sachdev V. Grief reaction, depression, anxiety, and coping of relatives after palliative patients' death in Thailand. PLOS ONE. 2022;17: e0276583. doi: [10.1371/journal.pone.0276583](https://doi.org/10.1371/journal.pone.0276583).

**Materials and methods**

*#39. At line 107, the fourth point of exclusion criteria, which are the “serious illnesses” that would preclude the participation in the study, and which are the differences between these “serious illnesses” and the conditions associated with depressive tendency stated at the fifth point of exclusion criteria?*

Response: In our study protocol, serious illnesses were disorders of consciousness and severe heart failure. We have revised the manuscript as follows.

(Line 115-116) (iv) disorders of consciousness or severe heart failure;

*#40. At line 126, please explain more deeply the statistic used for the calculation of sample size. What is the rationale behind the choice of repeat the measurements after 9 months and not, for example, after 12 months?*

Response: Details about the calculation of sample size, and the reason why the survey was conducted at 9 months have been mentioned as follows:

(Line 136) For multivariate logistic regression analysis, the size of the smaller category of the dependent variable should be at least 10 times the number of independent variables (28). Given the nature of the statistics, and approximately 50% prevalence of depressive tendencies in patients after PCI, the sample size was set to 80 or more to accommodate up to four independent variables (15,16).

(Line 133-135) The questionnaires were performed at baseline and again by mail at 9 months post-PCI, when patients were just beyond the high-risk period for target lesion revascularization (27).

Additional reference:

27.	Stolker JM, Cohen DJ, Kennedy KF, Pencina MJ, Lindsey JB, Mauri L, et al. Repeat revascularization after contemporary percutaneous coronary intervention: An evaluation of staged, target lesion, and other unplanned revascularization procedures during the first year. Circ Cardiovasc Interv. 2012;5: 772-782. doi: [10.1161/CIRCINTERVENTIONS.111.967802](https://doi.org/10.1161/CIRCINTERVENTIONS.111.967802).

**Statistical Analysis**

*#41. Lines 172 - 174, when explaining the division into four groups, please indicate which four groups are, for the sake of clarity, to avoid any misunderstanding. I suggest adding, at the end of the statistical analysis in the sentence “then the total SDS scores at baseline was compared with those at 9 months” the words:” for each group”, to better explain what you are doing.*

Response: We have added "for each group" at the end of the sentence (line 190).

**Results**

*#42. The results section contains a huge list of data in which it is easy to get lost, I recommend at the end of each section to make a small summary of the results obtained, even in tabular form, as the authors prefer, in order to fix the results in a more readable form.*

　Response: Following your advice, we have added explanations to each table. We believe these notes will help readers better understand the results of the study.

Table 2: Significant differences were observed in the followings: "Male/female," "Abandonment or Resignation," "Positive interpretation," and "Planning."

Table 3: In both models with adjustment, only "Planning" demonstrated an association with depressive tendencies at 9 months.

Table 4: Comparison of baseline coping strategy subscales between the group with depressive tendency at baseline but not at 9 months and the group with depressive tendency at both baseline and 9 months revealed significant differences in "Abandonment or Resignation," "Evasive thinking," "Positive interpretation," and "Planning."

Table 5: All variables except model 2 for evasive thinking were significant.

**Discussion and Conclusion**

*#43. I suggest dividing the discussion section into an initial 'introductory' part where there is a general discussion of what happened (line 288-291), then a separated discussion for each aim of the study (first aim: line 292-334; second aim: 335-352).*

Response: We have added subheadings to the Discussion section as follows:

(Line 317) Associations between depressive symptoms at 9 months and baseline characteristics

(Line 355) Coping strategies at the time of hospitalization related to improvement in depressive tendencies at 9 months

(Line 375) Depressive tendencies at 9 months and the influence of Type D personality during hospitalization

*#44. In lines 304-310 I would add, when talking about psychotherapy to improve coping strategies and to have an improvement in depressive tendency, that it is well known that even when diseases are cured, there are important sequelae in the well-being and sleep quality of formerly ill subjects (10.3390/ijerph21020129 ), the sleep quality factor can have a heavy influence on the maintenance or worsening of depressive tendency (10.1016/j.psychres.2020.113239). Thus, taking improved sleep hygiene into consideration, in addition to interventions aimed at improving the well-being of the subjects, may lead to improved prognosis outcomes. I would suggest including the two important papers I reported to define this aspect regarding sleep hygiene in the discussion and add the prospect of improved 'sleep hygiene in the conclusions as a means to achieve an improvement in depressive tendency and thus a better prognosis.*

Response: The manuscript was revised by adding importance of sleep disorders to the discussion section as follows.

(Line 348) Both current and resolved medical conditions, including CAD have been associated with insomnia and impaired well-being (48) and, life-changing events have been directly linked with sleep disturbances (49). Therefore, care treatment should also address sleep disorders, which have significant interactions with depressive tendencies and CAD (50,51). Further research is needed to develop instructional programs that address both cardiovascular diseases and sleep disorders.

Additional references:

48.	Menicucci D, Bastiani L, Malloggi E, Denoth F, Gemignani A, Molinaro S. Impaired well-being and insomnia as residuals of resolved medical conditions: Survey in the Italian population. Int J Environ Res Public Health. 2024;21: 129. doi: [10.3390/ijerph21020129](https://doi.org/10.3390/ijerph21020129).

49.	Umar A, Khan MS, Sehgal SA, Jafar K, Ahmad S, Waheed A, et al. Epidemiological studies of sleep disorder in educational community of Pakistani population, its major risk factors and associated diseases. PLOS ONE. 2022;17: e0266739, doi: [10.1371/journal.pone.0266739](https://doi.org/10.1371/journal.pone.0266739).

50.	Pandi-Perumal SR, Monti JM, Burman D, Karthikeyan R, BaHammam AS, Spence DW, et al. Clarifying the role of sleep in depression: A narrative review. Psychiatry Res. 2020;291: 113239. doi: [10.1016/j.psychres.2020.113239](https://doi.org/10.1016/j.psychres.2020.113239).

51.	Matsuda R, Kohno T, Kohsaka S, Shiraishi Y, Katsumata Y, Hayashida K, et al. Psychological disturbances and their association with sleep disturbances in patients admitted for cardiovascular diseases. PLOS ONE. 2021;16: e0244484. doi: [10.1371/journal.pone.0244484](https://doi.org/10.1371/journal.pone.0244484).
