# Supplementary material for: Coping strategies and changes in type D personality were associated with depressive tendency at 9 months after percutaneous coronary intervention
Source: PLoS One. 2025 Jan 13;20(1):e0316639. doi: 10.1371/journal.pone.0316639 (PMC11729923; doi:10.1371/journal.pone.0316639)
Supplement: Attachment — Submitted filename: Response_to_Reviewers.docx [file pone.0316639.s006.docx]

**Response to the Reviewers**

Thank you for reviewing our manuscript. We have revised the manuscript in accordance with the feedback provided.

**Response to Reviewer 1's review comments**

Thank you for your valuable feedback, we have revised the text accordingly. We have requested the translation company Editage to proofread the English. Our responses to your comments and the corresponding revisions in the manuscript are set out below.

*#1. Sample Size Justification:*

*While it is discussed the rationale for using a sample size of 80 patients, the choice of a 9-month follow-up rather than a more conventional 12-month period is still only briefly explained. Further elaboration on why this timeline was selected and how it may impact the generalizability of the findings would strengthen the study's robustness.*

　Response: Thank you for your comments regarding the choice of a 9-month follow-up. The reported prevalence of depression or anxiety in patients with coronary artery disease (CAD) was 24.3% and 23.1% at the 6-month and 12-month follow-up, respectively [28]. In a study investigating patients after myocardial infarction, the prevalence of depression was 37.7% and 37.2% at the 4-month and 12-month follow-up, respectively [29]. The prevalence of depression appeared to remain constant for up to 12 months, with a slight increase thereafter [28]. These reports suggest that the prevalence of depressive tendency is stable at 9 months, and that other factors may play a role in the psychopathology of depression beyond 12 months.

Based on these reports and considerations, we conducted our survey at 9 months post-PCI, when the depressive tendency was in a stable chronic phase. The text and corresponding citations have been revised. as follows:

(Lines 130–134) The reported prevalence of depression or anxiety in patients with CAD remains constant for up to 12 months [28, 29], increasing thereafter [28], suggesting that other factors could have an effect on the long-term psychological well-being of these patients. Therefore, the evaluation was conducted at 9 months, when patients were in a stable chronic phase of depressive tendencies.

Additional references:

28.	Palacios JE, Khondoker M, Achilla E, Tylee A, Hotopf M. A Single, One-Off Measure of Depression and Anxiety Predicts Future Symptoms, Higher Healthcare Costs, and Lower Quality of Life in Coronary Heart Disease Patients: Analysis from a Multi-Wave, Primary Care Cohort Study. PLOS ONE. 2016;11: e0158163. doi: 10.1371/journal.pone.0158163.

29. Lane D, Carroll D, Ring C, Beevers DG, Lip GY. The prevalence and persistence of depression and anxiety following myocardial infarction. Br J Health Psychol. 2002;7: 11-21. doi: 10.1348/135910702169321.

*#2. Handling of Missing Data:*

*Although you provided a table comparing respondents with complete and missing data, there is no clear explanation of how missing data was handled in statistical analysis. It remains unclear whether any statistical techniques were considered and why you chose exclusion over other methods.*

Response: Thank you for raising these issues. We have considered other methods of handling missing values, such as data imputation techniques. However, imputing missing values can introduce bias depending on the method used, which may affect the validity of the results. For example, simple imputation methods such as mean substitution can underestimate the variability in the data, while more complex methods such as multiple imputation require assumptions about the missing data mechanism that may not hold true. Taking into account these potential issues, we concluded that excluding cases with missing values would minimize the risk of bias and ensure the robustness of our findings.

We have added the following sentences to the manuscript:

(Lines 208–212) We did not to use other statistical techniques for handling missing values, such as data imputation methods, because imputing missing values could introduce bias depending on the method used, potentially affecting the validity of the results. Furthermore, there were no significant differences in the baseline characteristics between the 84 patients with complete values and patients with missing values.

*#3. Exploration of Limitations:*

*While you have some discussion of limitations, especially regarding the small sample size, other limitations, such as the potential biases introduced by self-reported measures, have not been fully explored. The study’s reliance on self-reporting for both depression and coping strategies could introduce reporting bias, which should be acknowledged more explicitly.*

Response: Thank you for raising this important point. We agree that potential biases may arise from self-reported measures. In particular, patients with depressive tendencies may experience reduced motivation or concentration, making reporting bias more likely to occur. We have added the following text:

(Lines 410–413) Sixth, self-reporting bias may be present in the design of this study, particularly among patients with depressive tendency who may have reduced motivation or concentration to complete the questionnaire.

*#4. Concluding Remarks:*

*The conclusion remains somewhat broad, focusing on the potential for personality-oriented interventions without clearly linking this to the specific findings of the study. Adding more concrete suggestions for clinical practice or future research based on the study’s findings could strengthen the practical implications of the study.*

Response: Thank you for your insightful comments. We agree have extensively revised the conclusion, as follows:

(Lines 416–423) This study demonstrated that the pre-discharge adoption of stress coping strategies and changes in type D personality were associated with the persistence or improvement of depressive tendencies in patients with CAD. Based on the association between personality and the psychopathology of depressive tendency, personalized feedback interventions targeting type D personality may offer novel approaches for delivering patient-centered care. In conclusion, personality-oriented interventions and/or education incorporating specific coping strategies may be effective in preventing and alleviating depression, which could, in turn, positively impact the prognosis of patients with CAD.

*#5. Conclusion:*

*You have made significant improvements, particularly in the areas of clarity, structure, and transparency. However, some refinements could be made. Overall, this manuscript is much more suitable for publication, but the minor revisions could further enhance its quality and impact.*

Response: We appreciate your continued support and positive feedback and hope that the revised version of the manuscript is suitable for publication.

*#6. References: Please, check the SPIRR-CAD trial reference. In your text you describe the study with reference number 48, later with another number (53). I suppose that this is an error because you have added further references recommended by another reviewer.*

Response: Thank you for raising this issue. We have replaced reference number “[48]” with “[55]” (Line 392).
