# Supplementary material for: Coping strategies and changes in type D personality were associated with depressive tendency at 9 months after percutaneous coronary intervention
Source: PLoS One. 2025 Jan 13;20(1):e0316639. doi: 10.1371/journal.pone.0316639 (PMC11729923; doi:10.1371/journal.pone.0316639)
Supplement: Attachment — Submitted filename: Response to Reviewers.docx [file pone.0316639.s007.docx]

**Response to the Reviewers**

Thank you for reviewing our manuscript. We have revised it in accordance with the feedback provided.

Response to Reviewer 1's review comments

Thank you for your valuable feedback. We have revised the text accordingly. We have also requested the translation company Editage to proofread the English. Our responses to your comments, along with the corresponding revisions in the manuscript, are outlined below.

#1. Sample Size Justification:

Reviewer #1: Most of my suggestions/concerns were addressed.

Only one aspect:

Page 7, line 130: The reported prevalence of depression or anxiety in patients with

131 CAD remains constant for up to 12 months [28, 29], increasing thereafter [28],

132 suggesting that other factors could have an effect on the long-term psychological well

133 being of these patients. The evaluation was therefore conducted at 9 months, when

134 patients were in a stable chronic phase of depressive tendencies.

From my point of view this is not logical. Why do you not assess after e.g. 24 months? It could be important to correlate your pre-assessment with long-term outcome with increased values for anxiety and depression.

**Response:** Thank you for your valuable comments. We agree with your suggestion and have deleted the relevant part of the text: "increasing thereafter [28], suggesting that other factors could have an effect on the long-term psychological well-being of these patients." We acknowledge that the lack of long-term psychological evaluations is a limitation of this study.

In this study, we evaluated the impact of PCI during hospitalization on patient psychology 9 months after discharge, as the aim was to assess patients in the early chronic phase. We have added three references [1,2,3] that reported the effects of cognitive behavioral therapy or psychological evaluations within 12 months.

Regarding the seventh limitation, we have clarified in the limitations section that the evaluation was conducted at a single time point, 9 months after discharge, and that long-term studies are needed to account for the potential impacts of other factors (e.g., life events independent of PCI) on later mental health outcomes.

We hope that the revised version of the manuscript is now suitable for publication.

**Additional references:**

1. Stafford L, Berk M, Reddy P, Jackson HJ. Comorbid depression and health-related quality of life in patients with coronary artery disease. J Psychosom Res. 2007;62: 401-410. doi: 10.1016/j.jpsychores.2006.12.009.

2. Goyal M, Singh S, Sibinga EM, Gould NF, Rowland-Seymour A, Sharma R, et al. Meditation programs for psychological stress and well-being: a systematic review and meta-analysis. JAMA Intern Med. 2014;174: 357-368. doi: 10.1001/jamainternmed.2013.13018.

3. Karyotaki E, Efthimiou O, Miguel C, Bermpohl FMG, Furukawa TA, Cuijpers P, et al. Internet-based cognitive behavioral therapy for depression: a systematic review and individual patient data network meta-analysis. JAMA Psychiatry. 2021;78: 361-371. doi: 10.1001/jamapsychiatry.2020.4364.

**Corrections from lines 131-134:**

“This study aimed to evaluate the psychological impact of PCI during hospitalization in the early chronic phase after discharge. Therefore, we conducted an evaluation at 9 months, referencing studies that reported psychological assessments within 12 months [30,31,32].”

**Addition of the seventh limitation to lines 411-414:**

“Seventh, the follow-up evaluation was conducted only at a single time point, 9 months after discharge. Long-term longitudinal studies that account for the potential impacts of other life events on subsequent mental health outcomes after PCI are needed.”
